# Technical Note: Defining cyclotron‐based clinical scanning proton machines in a FLUKA Monte Carlo system

**DOI:** 10.1002/mp.12701

**Published:** 2017-12-22

**Authors:** Francesca Fiorini, Niek Schreuder, Frank Van den Heuvel

**Affiliations:** ^1^ CRUK – MRC Oxford Institute for Radiation Oncology University of Oxford Oxford UK; ^2^ Provision Proton Therapy Center Knoxville TN USA

**Keywords:** cyclotron accelerated beams, FLUKA simulations, Monte Carlo treatment verification, proton therapy, RayStation TPS, treatment planning

## Abstract

**Purpose:**

Cyclotron‐based pencil beam scanning (PBS) proton machines represent nowadays the majority and most affordable choice for proton therapy facilities, however, their representation in Monte Carlo (MC) codes is more complex than passively scattered proton system‐ or synchrotron‐based PBS machines. This is because degraders are used to decrease the energy from the cyclotron maximum energy to the desired energy, resulting in a unique spot size, divergence, and energy spread depending on the amount of degradation. This manuscript outlines a generalized methodology to characterize a cyclotron‐based PBS machine in a general‐purpose MC code. The code can then be used to generate clinically relevant plans starting from commercial TPS plans.

**Methods:**

The described beam is produced at the Provision Proton Therapy Center (Knoxville, TN, USA) using a cyclotron‐based IBA Proteus Plus equipment. We characterized the Provision beam in the MC FLUKA using the experimental commissioning data. The code was then validated using experimental data in water phantoms for single pencil beams and larger irregular fields. Comparisons with RayStation TPS plans are also presented.

**Results:**

Comparisons of experimental, simulated, and planned dose depositions in water plans show that same doses are calculated by both programs inside the target areas, while penumbrae differences are found at the field edges. These differences are lower for the MC, with a *γ*(3%–3 mm) index never below 95%.

**Conclusions:**

Extensive explanations on how MC codes can be adapted to simulate cyclotron‐based scanning proton machines are given with the aim of using the MC as a TPS verification tool to check and improve clinical plans. For all the tested cases, we showed that dose differences with experimental data are lower for the MC than TPS, implying that the created FLUKA beam model is better able to describe the experimental beam.

## 1 Introduction

An increasing number of particle therapy facilities are nowadays functional with active pencil beam scanning (PBS) replacing passive scattered beam systems. This has been a substantial improvement in terms of flexibility for shaping the dose distribution,^1^, ^2^ but at the same time the commercially available treatment planning systems (TPS) had to start dealing with an increased number of complex calculations to determine the dose deposited in the treated volumes. Analytical dose calculations used in TPSs employ simplified algorithms, that have the advantage of being fast and can calculate many plans in a relatively short time. The disadvantage being their reliability in some conditions. Of particular concern is the dose deposition in highly inhomogeneous patient regions,^3^, ^4^ that is, in the presence of metal implants, thick bones, or cavities. The treatment of small volumes requiring the use of small fields,^5^ or the treatment of shallow regions requiring the use of range shifters and large air gaps are also not accurately modeled by commercial TPSs. In these cases, Monte Carlo simulations are a valuable instrument to obtain more reliable dose maps.^6^, ^7^ General purpose Monte Carlo (MC)‐based codes are also able to accurately calculate the dose outside high‐dose regions, providing better estimate of the dose to the healthy tissues and can provide dose deposited by various particles separately. Indeed, secondary particles such as neutrons, light ions, delta‐rays, x rays, and gamma photons can be tracked and by scoring them separately we can assess their radiobiological impact through LET,^8^ RBE^9^ or DNA damage conversion,^10^, ^11^ which might be important in assessing the quality of a particle treatment.

In the literature we can find many manuscripts reporting TPS‐MC comparisons^12^, ^13^, ^14^, ^15^ showing maximum differences ≤±10% for passively scattered proton therapy. Other studies^5^, ^16^ are focused on synchrotron‐accelerated PBS systems, for which the MC characterization is less convoluted, as most of the beam qualities (energy spread, lateral spread) are energy independent. Some newest commercial TPSs include a fast MC algorithm able to calculate more accurate dose maps than their PBS algorithm, however, they still do not include all the physics included in a general purpose MC. Moreover for most of them only dose can be calculated. This manuscript aims to outline a generalized and independent method to characterize a cyclotron‐based PBS machine in a general purpose MC code, and more particularly in FLUKA^17^, ^18^, ^19^ (version 2011.2c). To illustrate the method we characterized the beam accelerated at the cyclotron‐based Provision Proton Therapy Center (Knoxville, TN, USA) and presented the MC verification of phantom plans made with the RayStation TPS (version 5, RaySearch Laboratories ©).

## 2 Material and methods: Beam characterization

The Provision installation consists of an IBA cyclotron‐based equipment (Proteus Plus), which provides proton beam scanning technique up to a maximum energy of 230 MeV. Energies down to 98 MeV are obtained using a set of degraders at the exit of the accelerator. Energies down to 30 MeV are obtained using a range shifter placed at the end of the snout, which is in the vicinity of the patient. This results in a unique spot size, divergence, and energy spread depending on the amount of degradation. Two different commissioning data sets were used for the characterization:

- water phantom measurements were used to relate the experimental beam energy and energy spread to the simulated beam energy and energy spread. This was measured using a PTW Bragg Peak ion chamber;
- in air measurements, were used to determine the spot size and divergence of the beams as a function of the beam energy. A Lynx high‐resolution scintillator‐based sensor with size 30 × 30 cm^2^ was used.

In order to fully describe a clinical treatment machine via MC simulations there might be several options. Here, we opted not to simulate the entire beam line, but to define a virtual source placed at a specific position in the nozzle with spectral and spatial characteristics defined to fit the measured data. The details of the virtual source definition and the HU–material conversion are described in the following sections.

### 2.A. Virtual origin

**Figure 1:**
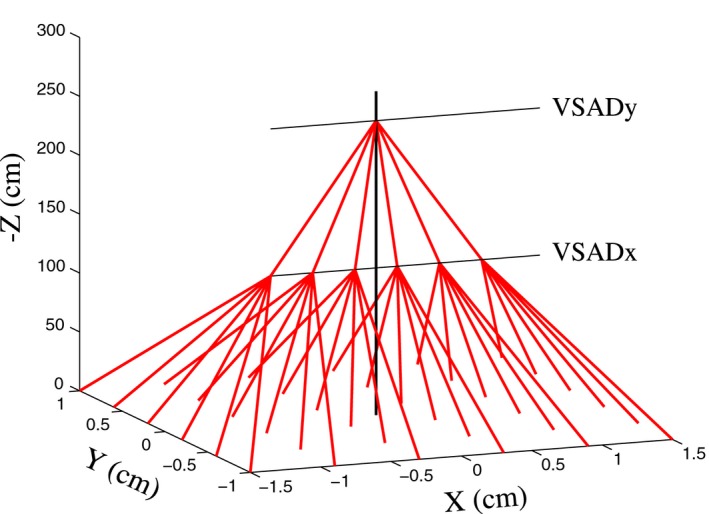
Schematic graph of the disposition of the virtual sources (VSAD_*x*_ and VSAD_*y*_) in the experimental and TPS setting representing the position of the focusing magnets *f*_*x*_ and *f*_*y*_ and the related beamlet final composite directions. [Color figure can be viewed at wileyonlinelibrary.com]

In the case of an IBA Proteus Plus machine, two different virtual source positions are given, one for the X axis and one for the Y axis. These are defined along the Z axis ([0,0,*Z*_*VSADx*_] and [0,0,*Z*_*VSADy*_], where [0,0,0] is the isocenter, see Fig. 1) and depend on the position of the effective focal points of the scanning magnets *f*_*x*_ and *f*_*y*_ which focus the beamlets along the X and Y axis, respectively. These virtual sources can be geometrically calculated using the experimental spot size of pencil beams at several distances from the isocenter and assuming that at the virtual sources the spot size on X (for *Z*_*VSADx*_) and Y (for *Z*_*VSADy*_) is zero. For the Provision beam, we found *Z*_*VSADx*_ = –193.5 cm and *Z*_*VSADy*_ = –231.5 cm. Since we decided not to simulate the focusing magnets in the MC, the best way to reproduce the experimental shape of the beamlets is to fix the MC source at the focal point closest to the isocenter and define the spot in X and in Y as it should actually be in that point. In this case, the real beam would be firstly focused in Y at *Z*_*VSADy*_, so at *Z*_*VSADx*_, where the MC source is placed, it would be already spread on the Y axis by the divergence in Y, while on the X axis it would be a point source: the resulting shape would be a line source. From *Z*_*VSADx*_ forward both divergences on X and Y affect the beam, which would then acquire an elliptic shape. This method effectively recreates the outcome of having two separate focal points without actually simulate the focusing magnets.

### 2.B. Lateral spread characterization

**Figure 2:**
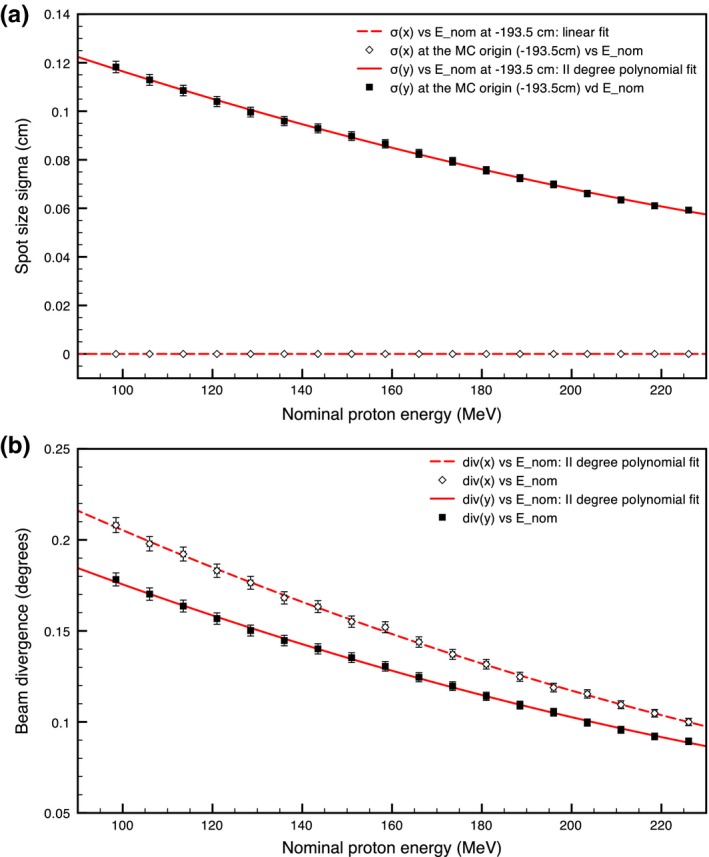
(a) Spot size (standard deviation of the Gaussian distribution) and (b) divergence characterization for the X and Y axes. [Color figure can be viewed at wileyonlinelibrary.com]

The experimental data collected in air at various distances (–20 ≤ z_*i*_(cm) ≤ 20, with the snout placed at –50 cm) from the isocenter (z_*i*_ = 0 cm) are modeled as Gaussian distributions parametrized by a mean and a standard deviation in the X and Y axes (Σ_*x*,*i*_ and Σ_*y*,*i*_). This parametrization changes with the beam nominal energy, due to the varying thickness of the degrader at the exit of the accelerator, while the anisotropic nature of the beam spots is due to the different effective focal positions of the scanning magnets. The spot size, *σ*_*x*_(*E*) and *σ*_*y*_(*E*), and divergence, *div*_*x*_(*E*) and *div*_*y*_(*E*), of the commissioning beamlets at the chosen MC origin can be geometrically calculated assuming that the broadening of the beam in air is mainly due to the beam divergence (maximum experimental depth in air = 70 cm). The results are given in Fig. 2. Polynomial representations of *σ*_*x*_(*E*), *σ*_*y*_(*E*), *div*_*x*_(*E*) and *div*_*y*_(*E*) are then used in the simulation initialization to avoid the use of look‐up tables. As already explained in Section 2.A, the beam at the origin is modeled to represent a line source with a 2D Gaussian distribution characterized by a standard deviation in the X and Y axes (*σ*_*x*_(*E*) = 0 and *σ*_*y*_(*E*) given by the polynomial fit of the curve in Fig. 2(a)) to which a divergence (*div*_*x*_(*E*) and *div*_*y*_(*E*), respectively) is applied to accurately describe the elliptic shape of the experimental beams. As can be seen from the plots the divergence on the X axis is slightly larger than the divergence on the Y axis, while the opposite occurs for the spot *σ*. This results in a circular spot at the isocenter and slightly elliptical before and after.

### 2.C. Energy and energy spread characterization

We used the depth dose deposition commissioning dataset acquired in water to determine the beam energy characterization. We recreated the experimental setting in FLUKA and scored the dose deposited. All particles contributing to the dose deposition were considered in this simulation as in all of the other ones used for beam characterization.

**Figure 3:**
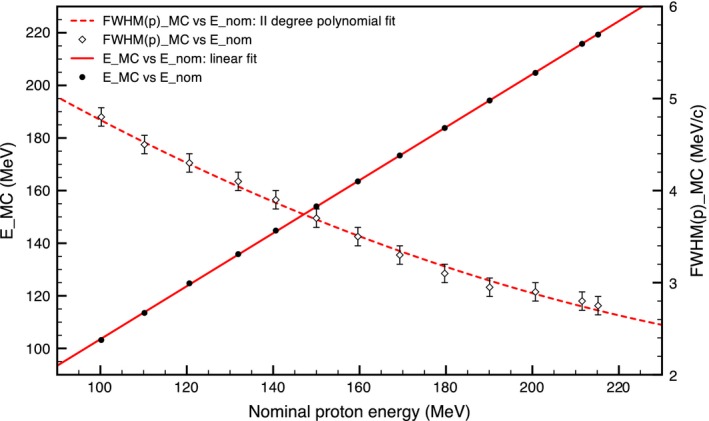
Full line (left hand‐side axis): relationship between the beam nominal energy and the simulation energy. The uncertainty for the simulated energies is below 1 MeV and therefore not visible. Dashed line (right hand‐side axis) relationship between the nominal energy and the momentum FWHM. [Color figure can be viewed at wileyonlinelibrary.com]

The beamlet energy spectrum at the source is modeled as a Gaussian distribution defined by a mean energy and a spread expressed in terms of the momentum full width half maximum ($FWHM\;=\;2\sqrt{2ln2}\;\sigma$). The peak position is determined by the value of the mean energy, while the spread is reflected in the peak/plateau ratio. In the simulation, the mean energy and spread required to obtain the same experimental depth‐dose depositions are found by varying both quantities exhaustively and sequentially optimizing them for the peak position and the peak/plateau ratio. To avoid using lookup tables, the relationships nominal energy — simulated energy and nominal energy — simulated momentum spread were characterized using polynomial fit functions. Both relationships are shown in Fig. 3. The reason behind the fact that *E*_*MC*_ and *E*_*nom*_ have different values, but effectively represent the same beam with the same range, is that they have been named using different definitions.

**Figure 4:**
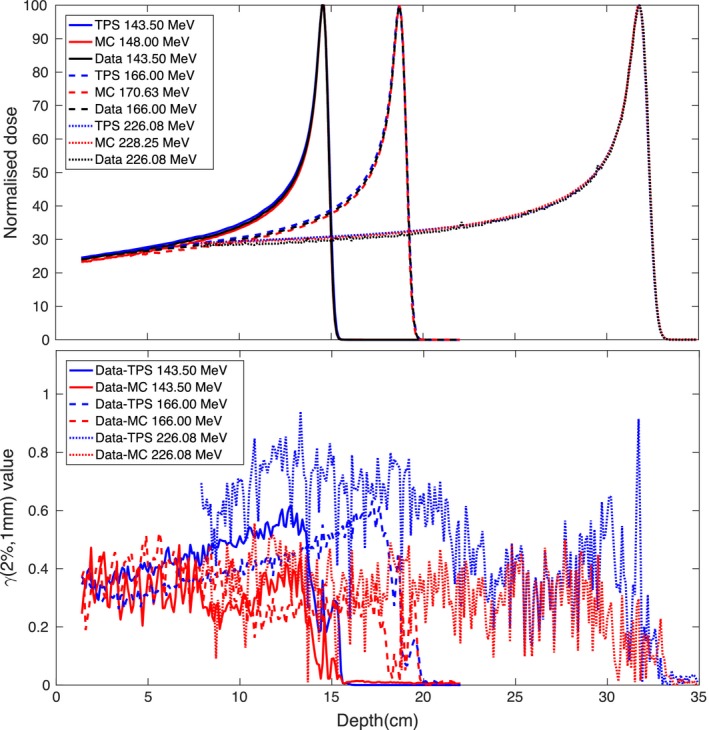
Top: depth–dose curves from FLUKA, RS and experiment acquired in water. The plotted doses are the normalized average doses calculated or measured in the cylindrical region experimentally occupied by the PTW chamber. Bottom: *γ*(2%, 1 mm) analysis results for the data–MC and data–TPS comparison for the above energy beams. [Color figure can be viewed at wileyonlinelibrary.com]

Once the energy characterization was obtained, we compared the depth dose depositions produced with FLUKA with those produced with RayStation (RS). The same geometrical setup was used in both programs. Some of the comparisons, which also include the experimental data and related gamma analysis, *γ*(2%,1mm), used as a quantitative method to compare the distributions for dose difference and spatial discrepancies,^20^ are shown in Fig. 4. As can be seen for all the considered cases the *γ*(2%,1 mm) values are below 1 and more specifically, the values for the data–MC comparison are lower than those calculated for the data–TPS comparison. The *γ* analysis was performed using as reference the experimental data and as evaluation the TPS or MC data. The bin size for all the data was 1 mm along the depth and the percentages were global.

### 2.D. Monitor unit calibration

A monitor unit calibration is required to determine the number of protons to use in the simulation to obtain the same delivered doses. Monitor units (MU) are an indirect measurement of dose derived from the charge deposited in a chamber in the snout. The accelerator will only deliver as many particles as the amount needed to reach the number of MUs requested by the TPS, for which an MU to dose‐to‐water calibration was performed during commissioning. Therefore, they depend on the beam energy and the calibration can be treated as a function relating the nominal energy to the number of delivered protons per MU planned. We defined this quantity k(E).

The experimental MU calibration for the TPS was carried out relating the experimental dose measured at a specific depth in the plateau region of a 10.5 × 10.5 cm^2^ field to the MU calculated by the TPS to deliver that dose. As that calibration was very accurate, in this work we decided to use the calibrated TPS MUs to find the number of protons needed to have the same TPS doses (and so same experimental doses) and opted to use a methodology based on total energy deposition. In fact, by using a phantom large enough and a comparable scoring volume, we were able to calculate the total energy deposition in the TPS and MC. We want to stress that this is not the only method to obtain a MU to number of protons calibration, other methods can be find in the literature.^21^ Let *D*_*TPS*_(*E*_*TPS*_) be the TPS‐integrated total dose and *d*_*MC*_(*E*_*MC*_) the simulated integrated dose deposited per unitary initial proton. The number of protons is then given by:

(1) $$N_{p}\left(E_{\mathrm{MC}}\right)\;=\;\frac{D_{\mathrm{TPS}}\left(E_{\mathrm{TPS}}\right)}{d_{\mathrm{MC}}\left(E_{\mathrm{MC}}\right)}.$$

with *E*_*TPS*_ the nominal energy given in the TPS RTplan file (which is assumed to be the same as the experimental one, but with the TPS label we want to stress that the energies are taken from an RTplan file) and *E*_*MC*_ the mean energy required in FLUKA to have the same range. The relationship between *E*_*TPS*_ and *E*_*MC*_ is given by the fit of the energy data in Fig. 3. As the MU calibration in the TPS is already defined, then *D*_*TPS*_(*E*_*TPS*_) = *d*_*TPS*_(*E*_*TPS*_) *MU*(*E*_*TPS*_), hence:

(2) $$N_{p}\left(E_{\mathrm{MC}}\right)\;=\;\frac{d_{\mathrm{TPS}}\left(E_{\mathrm{TPS}}\right)MU\left(E_{\mathrm{TPS}}\right)}{d_{\mathrm{MC}}\left(E_{\mathrm{MC}}\right)}.$$

**Figure 5:**
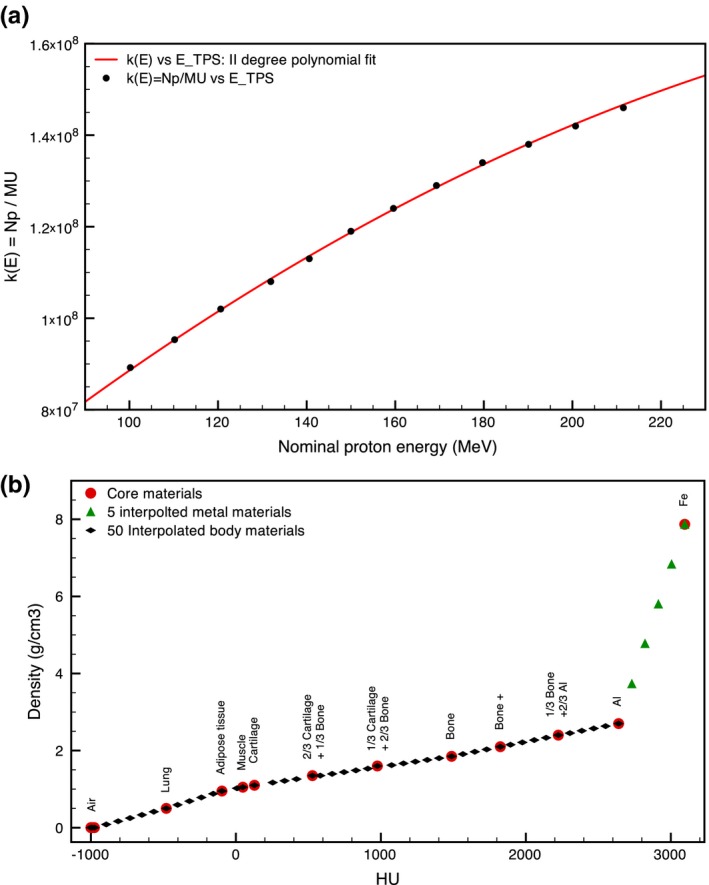
(a) MU calibration: k(E) is the parameter that multiplied by the MU of each energy beamlet gives the number of protons to simulate in that energy beamlet to have the same planned dose. The uncertainty of the k(E) data is of the order of 10^3^ and therefore not appreciable in this plot. (b) HU‐density conversion applied in RS to describe the body tissues and metal implants: 10 core materials plus Al and Fe (red circles), 50 interpolated body materials (black diamonds), and 5 interpolated metals (green triangles). [Color figure can be viewed at wileyonlinelibrary.com]

The energy calibration factor *k*(*E*_*TPS*_) is then defined as:

(3) $$k\left(E_{\mathrm{TPS}}\right)\;=\;\frac{d_{\mathrm{TPS}}\left(E_{\mathrm{TPS}}\right)}{d_{\mathrm{MC}}\left(E_{\mathrm{MC}}\right)}$$

which does not depend on the MU used in the plan but only on the beam nominal energy. The MUs used for each energy beamlet can be extracted from the TPS RTplan. For this calibration, we used simple pencil beams, one for each nominal energy and one per plan, so that in each RTplan there was only one single *MU*(*E*_*TPS*_) value to consider. We want to stress that, even if we know that there might be differences in the TPS and MC dose depositions mainly related to lateral scattering, by using the integral total dose to find the MU calibration, we get rid of the scattering issue, as we are not considering a dose in a particular point, but the total energy deposition which only depends on the initial beam spectrum and crossed material. We fitted the found *k*(*E*_*TPS*_) using a second order polynomial function, see Fig. 5(a).

At this point, every time we read an RTplan and have an MU value related to an energy beamlet, we know how many protons to consider in that beamlet by using: *N*_*p*_(*E*_*MC*_)_*i*_ = *k*(*E*_*TPS*_)*MU*(*E*_*TPS*_)_*i*_, where *i* represents the specific beamlet.

### 2.E. HU to proton stopping power calibration

In our FLUKA implementation, we based the CT to material conversion on the conversion used in RS. This is because both programs do not use tabulated stopping powers, but rely on the Bethe‐Bloch expression, with the due corrections, to calculate the *dE*/*dx*. For both a one‐to‐one conversion is needed from Hounsfield Units (HU). To determine this relationship a stoichiometric method based on mass density, composition, and ionization energy of 50 fixed materials was used. As explained in the RS reference manual, also for FLUKA we start from 10 established core materials which are expected to be found in a patient, with the addition of aluminum (Al) and iron (Fe), and for which HU, density, material composition, and mean ionization energy are defined (properties taken from ICRU49, ICRU44, ICRP23 reports). By linear interpolation we then calculate density, composition, and ionization energy for 50 body materials and additional five materials as a mixture of Al and Fe to account for high‐density metal implants [see Fig. 5(b)]. From the RS manual we can read: “Because of the relatively slow dependence of the relevant proton and electron quantities (e.g., mass stopping power) on the elemental composition, 50 fixed materials have been shown to be sufficient for patient like geometries. The material closest in mass density will be associated with a CT‐based voxel” (RayStation 5 Reference Manual, Section 2.D). In FLUKA, each of the 50 materials was identified by the average HU value of the covered HU range used in RS (conversion implemented in the materials.inp script), but the maximum and minimum density in the range were also provided in order to correct the calculation of the dE/dx (body.mat script).

### 2.F. Simulation initialization

The determined conversions, nominal energy → simulation energy, nominal energy → momentum spread and nominal energy → spot size and divergences, have been implemented in the FLUKA source file. Anytime an RTplan file is used to extract the beam information from the related TPS plan, the correct initial energies, momentum spread, spot size, and divergence to be used at the origin are calculated by FLUKA during the simulation initialization. The RTplan also includes the positions the beamlets have at the isocenter: the determination of the direction cosines calculated from the virtual origin to the position at the isocenter is also made in the source. The only conversion to be made before the simulation initialization is the conversion MU to number of protons, which in our case is done via another set of scripts using the MU calibration found in Section 2.D. These scripts read the RTplan and create a simpler ASCII file where only the information needed by FLUKA to initialize the simulation are present: number of fields, number of beamlets in each field, their energy, their number of particles, and their position in X and Y at the isocenter. Since it is usually not possible, nor even useful, to run the exact number of particles used in a plan (usually of the order of 10^10^−10^12^ protons, while we only need a number of events high enough to reduce the statistical error) the number of particles per energy in the ASCII file is considered by FLUKA as a weight, so that the particle ratio for each energy beamlet is kept unvaried. At the end of the simulation, by multiplying the calculated doses (in FLUKA given in GeVg^−1^/proton or Gy/proton) by the total number of particles in the ASCII file, it is possible to obtain the total deposited dose.

## 3 Results: Phantom verification

**Figure 6:**
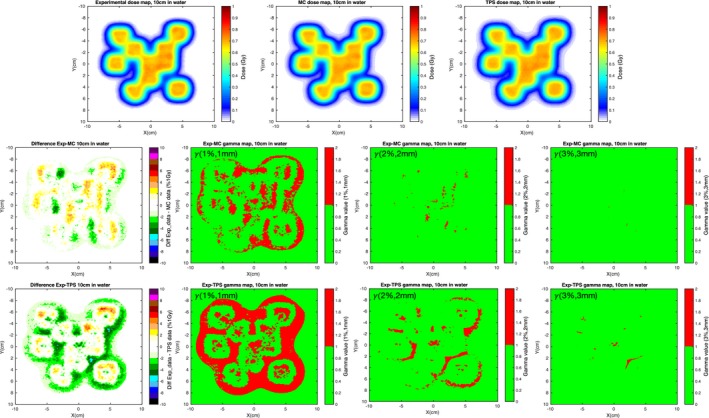
10 cm deep detection results. Top, left to right: 2D dose maps for experiment, MC and TPS. Center, left to right: 2D difference dose, *γ*(1%,1*mm*), *γ*(2%,2*mm*) and *γ*(3%,3*mm*) maps for data–MC. Bottom, left to right: 2D difference dose, *γ*(1%,1*mm*), *γ*(2%,2*mm*) and *γ*(3%,3*mm*) maps for data–TPS. [Color figure can be viewed at wileyonlinelibrary.com]

**Figure 7:**
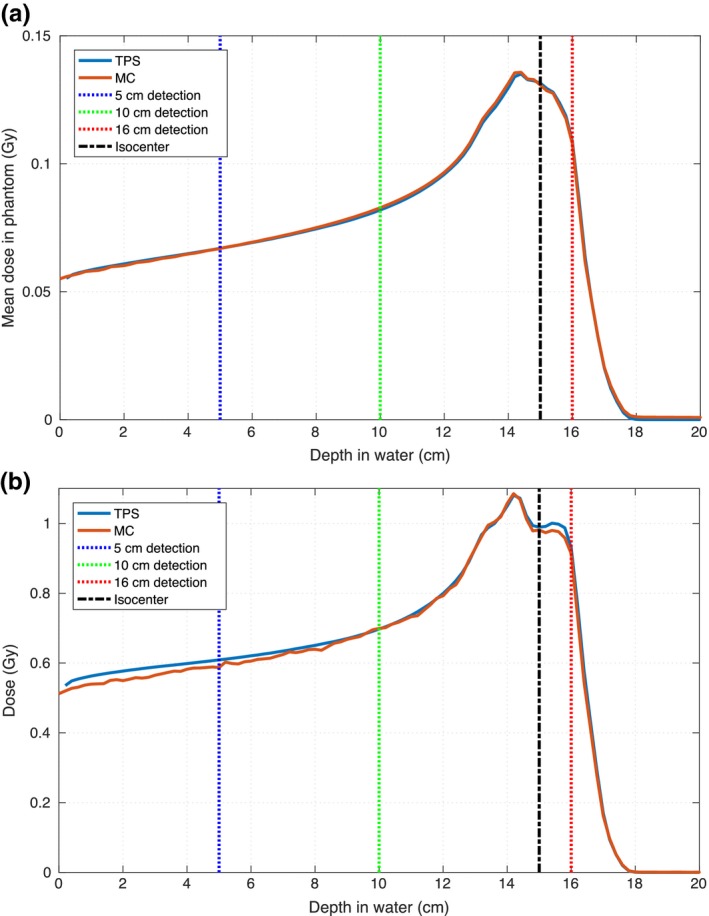
a) Overall depth‐dose deposition in the entire phantom and (b) depth‐dose deposition on the beam axis for MC and TPS. The depths where the experimental profile measurements were taken and the isocenter position are also shown. [Color figure can be viewed at wileyonlinelibrary.com]

We have already established that RS and FLUKA created comparable integral depth–dose depositions (see Fig. 4) for single pencil beams. In this chapter, we also discuss the lateral profile mainly dominated by lateral scattering. We used a plan involving several energy spots delivering 1 Gy to several different 1 × 1 × 1 cm^3^ targets scattered in a 12 × 12 cm^2^ area in a water phantom. The TPS and MC dose were scored in the entire phantom, while for the comparison with the experimental dose, dose maps were recorded at three depths in water using the Lynx and Matrix‐PT detectors. The isocenter was 15 cm deep in water (snout at 50 cm and phantom starting at 15 cm), while the three detector positions were 5, 10, and 16 cm deep. These depths correspond to 60 and 70% of the prescribed dose (D_1*Gy*_) in the plateau region for the first two positions, and 90% of D_1*Gy*_ in the tail region for the third (no dose can be measured beyond 18 cm, see Fig. 7). The Lynx detector, while providing signal maps with a high spatial resolution, it does not provide absolute doses. For this reason, we used it in conjunction with a Matrix‐PT chamber in order to convert the Lynx signal maps to dose maps. The average signal from the inner voxels of the target at the isocenter from the Lynx signal maps were firstly normalized to 1 and then multiplied by the average dose the Matrix‐PT maps provided for the voxels in the same target. This gave us a conversion factor signal to dose for each one of the three considered water depths, so that we could compare the high‐resolution experimental dose maps to the TPS and MC dose maps. A total of 3.6 × 10^8^ source protons were simulated, which gave us a statistical error lower than 1.5% in the target volumes and lower than 5% for doses larger than 5% of the prescribed dose (average CPU time per primary proton, 4 × 10^−3^ s, run on a 20 core‐computer). We compared the experimental, planned, and simulated dose profiles for these three depths. In order to perform an exhaustive comparison analysis, for all depths we calculated the difference dose maps, voxel by voxel, and the gamma analysis for data–MC and data–TPS. Some of these results are shown in Fig. 6 for the 10 cm deep detection. The other results, including the minimum and maximum values of the difference maps, and the percentage of passing voxels for the performed *γ* analysis, 1%–1 mm, 2%–2 mm and 3%–3 mm, are given in Table 1. The dose threshold for *γ* was 0 Gy on the experimental dose: for the comparison with TPS and MC we are considering all of the voxels with a non‐null dose in the experimental dose maps. While differences can be seen for both MC and TPS with similar minimum and maximum difference values (<±8% of D_1*Gy*_), the amount of voxels with a non null difference is higher for data–TPS: see first graphs on the left in the center and bottom row in Fig. 6. The same is true for the *γ* analysis, where for all the considered depths the amount of voxels passing the criteria is higher for the data‐MC case. From the table we see also that in both cases the agreement between measured and calculated doses decreases with increasing depth in water. This is due to small initial differences in the lateral profile propagating into the phantom. As shown in the *γ* maps, most of the non passing voxels are situated in regions where the measured dose is already low, that is, penumbra region outside the target volumes, so that by increasing the threshold on the experimental voxel dose (20% of the prescribed dose is an often used value) the number of passing voxels increases for both MC and TPS.

**Table 1:** Results of the analysis for all the detection depths and for both comparison data–MC and data–TPS

		Diff (% of 1 Gy)		% of non null voxels passing *γ* analysis		
	Depth (cm)	min	max	*γ*(1%, 1 mm)	*γ*(2%, 2 mm)	*γ*(3%, 3 mm)
data‐MC	5	−6.88	5.40	78.06	97.61	99.69
	10	−5.43	5.39	74.95	98.61	99.97
	16	−7.98	6.86	43.00	82.22	97.95
data‐TPS	5	−5.30	6.50	68.78	95.31	99.33
	10	−5.98	6.19	49.53	90.27	99.35
	16	−7.41	8.36	34.19	70.57	94.20

In Fig. 7 we also show the comparison between MC and TPS for the depth dose deposition, where in (a) is the overall average energy deposited along the phantom and on (b) the depth dose along the beam axis. While the graph in (a) shows that the total amount of deposited energy is, as expected, the same for both codes, the one in (b) shows again that there are differences in the local deposition of dose, mainly due to the way the two codes handle the lateral scattering.

## 4 Discussions and conclusions

In this manuscript, we outlined the steps to create a cyclotron accelerated PBS beam model in FLUKA starting from the commissioning measured data and MU calibration data available in the TPS. The beam modeled in this study was the proton beam cyclotron accelerated at the Provision Proton Therapy Center and the chosen TPS was RayStation, but other beams and other TPSs can be considered without any reason for changing the method. In order to show that the FLUKA representation of the Provision beam model was accurate, we compared experimental, planned, and simulated dose distributions for a plan involving several beam energies and a nonregular field shape. The results are shown in Figs. 6, 7 and Table 1, where we can see that the FLUKA‐simulated dose maps represent better the experimental ones. From the difference maps we can see that RS overestimates the lateral dose outside the targets, meaning that, with respect to FLUKA, the RS lateral beam profile is less accurately described. This might not only be partly due to the beam characterization in the model describing the facility in the TPS but also due to the actual lateral scattering algorithm used in RS to describe the beam behavior. In fact, starting from the original experimental Gaussian parameters, the TPS calculates a superposition of 19 Gaussian distributions (1 at the center, and 6 and 12 positioned at two concentric circles around the central spot), creating a slightly more spread out lateral distribution (refer to the RS 5 Reference Manual, section 5.B.1). The MC instead does not use any particular prefixed distribution, but follows particle by particle accounting for the different phenomena that might happen according to the known scattering theories (refer to the FLUKA manual, “FLUKA: a multi‐particle transport code”, section 1.B.1). This includes the production of secondary particles, such as photons (both X and *γ* rays), electrons (both low and high energy ones), light ions, neutrons… These are not entirely and explicitly taken into account in the TPS algorithm, but their dose deposition is considered as a deviation from the central Gaussian dose distribution of the beam core.^22^

Apart from this, we can still see, that not even the MC dose distributions are perfect, but for all the depths taken under consideration, which are representative of the beam behavior, the *γ*(3%,3 mm) index is never below 95%, threshold usually considered clinically.^23^ This implies that the created FLUKA model is better able to accurately describe the experimental beam.

Once the FLUKA beam model is benchmarked, it can be used as a treatment verification tool for clinical plans, with which we can check the plans and possibly correct them in the case the MC showed considerable different dose depositions. This would possibly reduce toxicity to healthy tissues and risk of secondary malignancies while increasing tumor control. In addition other radiobiologically relevant quantities could be scored, that is, LET, RBE, DNA damage, giving us an ulterior way to assess the quality of a particle treatment.

Finally, we want to remark that the recently added new functionalities of the FLUKA graphical interface, Flair,^19^ are not yet suitable to be used for cyclotron accelerated clinical PBS beams without custom modification, that is, coding, as they do not expect initial beam characteristics to change with the nominal beam energy.

## Conflicts of interest

The authors have no relevant conflicts of interest to disclose.
